# A Real-World Comparison of Apixaban and Rivaroxaban in Obese and Morbidly Obese Patients With Nonvalvular Atrial Fibrillation

**DOI:** 10.1177/08971900231202643

**Published:** 2023-09-15

**Authors:** Kevin T. Burnham, Tianrui Yang, Jessica Wooster

**Affiliations:** 1 Department of Pharmacy, 10591 Methodist Health System, Dallas, TX, USA; 2 Fisch College of Pharmacy, 12347 The University of Texas at Tyler, Dallas, TX, USA

**Keywords:** nonvalvular atrial fibrillation, anticoagulants, obesity, retrospective cohort study, apixaban

## Abstract

**Background:** Contemporary guidelines for managing nonvalvular atrial fibrillation (NVAF) include apixaban and rivaroxaban as first-line anticoagulation treatment options. Minimal guidance is available regarding selecting anticoagulants for patients with class I-III obesity. **Objective:** This study aims to evaluate the comparative effectiveness and safety of apixaban and rivaroxaban in both obese and morbidly obese patients with NVAF. **Methods:** A retrospective cohort study was conducted at an outpatient cardiovascular clinic after Institutional Review Board approval. Patients were eligible if they were ≥18 years of age, had a BMI ≥30 kg/m^2^, and took apixaban or rivaroxaban for NVAF for ≥3 months. The primary endpoint was the composite rate of stroke, transient ischemic attack (TIA), myocardial infarction (MI), or presence of atrial thrombosis. Bleeding events were evaluated as the primary safety endpoint. **Results:** Combined, the cohorts consisted of 303 obese or morbidly obese patients. The primary composite endpoint occurred in 3.8% of patients taking apixaban and 1.7% of patients taking rivaroxaban (*P* = .28). Both clinically relevant, non-major and major bleeding occurred more often in the apixaban arm, but this difference was not statistically significant; however, bleeding risk may have been skewed due to differences in baseline characteristics. **Conclusion and Relevance:** For obese and morbidly obese patients prescribed either apixaban or rivaroxaban for NVAF, rates of stroke, TIA, MI, and atrial thrombosis did not differ. The preferred DOAC for patients with class I-III obesity remains elusive, but current data points to a patient-centered approach for anticoagulant selection.

## Introduction

Atrial fibrillation (AF) places patients at risk for systemic thromboembolic events, namely ischemic stroke. Apixaban and rivaroxaban are direct oral anticoagulants (DOACs) that have shown non-inferiority to warfarin for the prevention of stroke and systemic thromboembolic events in patients with nonvalvular atrial fibrillation (NVAF).^1,2^ These 2 DOACs, along with dabigatran and edoxaban, are now recommended over warfarin for NVAF in current guidelines.^3^ Specific recommendations regarding anticoagulant choice in obese patients are still lacking. Of the anticoagulants used for NVAF, only warfarin has been shown to require higher daily doses with increasing body weight and required monitoring allows for patient-specific dosing irrespective of body habitus.^4^ In comparison to warfarin in pivotal phase 3 trials and some observational studies, apixaban and rivaroxaban did not seem to have compelling difference in efficacy in obese populations.^1,5,6^ In phase 3 studies comparing these DOACs to warfarin in NVAF, the rates of stroke and systemic embolism were 1.7 vs 2.2% per year for the rivaroxaban/warfarin comparison (.79 [.66-.96] *P* < .001) and 1.27 vs 1.6% per year for the apixaban/warfarin comparison (.79 [.66-.95] *P* = .01); however, both studies included a broad range of patient weights, which were not compellingly inclusive of obese patients.^1,2^ Guidance statements, provided by the International Society on Thrombosis and Haemostasis (ISTH), recommend that DOACs are avoided in patients with a BMI ≥40 kg/m^2^ or with a weight of ≥120 kg.^7,8^ This guidance statement is based on insufficient clinical evidence to support their use and pharmacokinetic (PK) changes seen with some DOACs.^7^ In part to their recommendations, they recommend the use of any DOAC for use in patients with weights up to the aforementioned thresholds. Appropriate selection of anticoagulants for obese patients is needed to maximize effectiveness while preventing major bleeding events.

In a PK study that included healthy patients who weighed ≥120 kg and BMI ≥ 30 kg/m^2^, maximum apixaban concentration (Cmax) was 31% lower (90% CI 18%–41%), and the area under the curve (AUC) was 23% lower (90% CI 9%–35%) in the obese group compared to reference group who weighed between 65 to 85 kg.^9^ Significant differences in Cmax and AUC were not seen in healthy patients weighing ≥120 kg who were given rivaroxaban compared to the reference group weighing between 70 to 80 kg.^10^ Although, 1 PK study has shown a potential 16% decrease in AUC and 17% for simulated patients of 150 kg with fixed doses of rivaroxaban.^11^ The clinical implication of this difference is unknown, and a comparison of clinical effectiveness between these agents in obese patients is required to uncover potential differences in efficacy between DOAC agents.

Lower-quality studies, including single-center observational studies, evaluating the safety and efficacy of DOACs compared to warfarin have been conducted that include morbidly obese patients (BMI ≥ 40 kg/m^2^).^5,6,12^ These studies all compare warfarin to DOACs collectively and do not include obese patients with BMI between 30 to 40 kg/m^2^. Previous studies do not focus directly on the intercomparison of DOACs to 1 another and do not offer insight into the PK differences. Our goal in conducting this study is to compare the efficacy and safety of apixaban to rivaroxaban for patients with BMIs ≥ 30 kg/m^2^. To our knowledge, there is no existing study that directly compares apixaban to rivaroxaban in patients with NVAF and BMIs ≥ 30 kg/m^2^.

## Patients and Methods

### Study Design and Endpoints

A retrospective chart review was conducted on patients who had been prescribed either apixaban or rivaroxaban from a single outpatient cardiovascular clinic. Patients started on either DOAC from October 2014 to January 2021 were evaluated for study inclusion. Inclusion criteria included patients 18 years or older, a diagnosis of NVAF, prescribed apixaban or rivaroxaban for ≥3 months, and having a BMI ≥ 30 kg/m^2^. The definitions for obesity (≥30 kg/m^2^) and morbid obesity (≥40 kg/m^2^), used for the study inclusion and delineation of baseline characteristics, was based on the World Health Organization’s definitions.^13^ Patients were excluded if they received DOACs for indications other than preventing stroke or embolism in NVAF. This study protocol was reviewed and approved by the institutional review board.

The cardiovascular clinic is part of a health system that includes several tertiary inpatient facilities with shared electronic health records. Once eligible patients were identified, records from follow-up clinic visits and inpatient charts were evaluated to define patient characteristics and locate relevant endpoints. An evaluable period included information from when a patient was prescribed rivaroxaban or apixaban until medication discontinuation. No further information was assessed once a patient stopped taking the studied DOAC or switched to another anticoagulant. Baseline characteristics were determined from the index outpatient visit where DOAC use was first identified.

The primary endpoint evaluating DOAC effectiveness was the composite rate of stroke, transient ischemic attack (TIA), myocardial infarction (MI), or presence of atrial thrombosis. For secondary endpoints, components of the primary endpoint were evaluated individually. Clinically relevant, non-major bleeds and major bleeding events, as defined by ISTH, were assessed as the primary safety endpoint.^14^ Each suspected outcome was evaluated and confirmed or refuted by all study investigators. All-cause mortality was considered as a secondary exploratory endpoint.

### Statistical Analysis

Data’s normalcy was tested with the Shapiro-Wilk test. Differences between baseline characteristics and endpoints were tested using chi-square and independent t-tests. Non-normally distributed data (i.e., age, CHA₂DS₂-VASc scores, and HAS-BLED scores) were tested with Mann-Whitney U tests and transformed for modeling. Logistic regression was run to identify variables significantly related to components of the primary endpoint, bleeding and death, and significantly different demographic variables between groups were controlled for as covariates. No adjustments for multiplicity were conducted. Kaplan-Meier’s were not calculated due to the small number of events observed.

## Results

Three hundred and thirty three patients were included in the study. Demographic information is presented in Table 1. There were 55 excluded because they had not been on the DOAC for ≥3 months (25; 45.5%), did not have a BMI ≥ 30 kg/m^2^ on index visit (25; 45.5%), were taking a different anticoagulant (3; 5.5%), or complete medical records were not available (2; 3.6%). Both cohorts were balanced in most characteristics, but significant differences were seen in alcohol use, HAS-BLED scores, and prior bleeding events, which were all more common in the apixaban arm. Renal disease was more prevalent in the rivaroxaban arm.

**Table 1.** Baseline Characteristics.

	Rivaroxaban (n = 121)	Apixaban (n = 212)	*P*-value

Age, years (mean ± SD)	69.5 ± 10.4	71.4 ± 10.0	.10
Age ≥ 65 (n, %)	86 (71.1%)	159 (75.0%)	.44
Male (n, %)	66 (54.5%)	124 (58.5%)	.49
“Ongoing DOAC treatment” (n, %)	100 (82.6%)	174 (82.1%)	.81
Caucasian (n, %)	110 (90.1%)	194 (91.5%)	.85
Hispanic (n, %)	2 (1.7%)	2 (.9%)	.57
Drinks alcohol^a^ (n, %)	12 (9.9%)	42 (19.8%)	.02
Active smoker (n, %)	10 (8.2%)	16 (7.5%)	.51
Prescribed ≥1 concomitant medication associated with bleeding	62 (51.2%)	131 (61.8%)	.06

BMI, kg/m^2^ (mean ± SD)	38.3 ± 7.5	37.2 ± 6.9	.19
Morbidly obese (n, %)	32 (26.4%)	61 (28.8%)	.65
BMI of morbidly obese group, kg/m^2^ (mean ± SD)	48.44 ± 7.5	46.1 ± 6.2	.11

CHA₂DS₂-VASc score (mean ± SD)	4.2 ± 1.8	4.2 ± 1.8	.48
HAS-BLED score^a^ (mean ± SD)	1.7 ± 1.0	2.03 ± 1.1	.01

Morbidities (n, %)			
CHF	60 (49.6%)	103 (48.6%)	.87
DM-II	44 (36.4%)	87 (41.0%)	.40
HTN	111 (91.7%)	187 (88.2%)	.31
Uncontrolled hypertension	28 (23.1%)	67 (31.6%)	.10
Prior stroke	12 (9.9%)	17 (8.0%)	.56
Vascular disease	78 (64.5%)	118 (55.7%)	.12
Renal disease^a^	26 (21.5%)	26 (12.3%)	.03
Liver disease	3 (2.5%)	5 (2.4%)	.95
History of bleeding^a^	5 (4.1%)	24 (11.3%)	.03

There were no statistical differences in BMI, or weight classes between study branches. Overall, obese patients’ BMI ranged 27.8-39.8 kg/m^2^ (IQR: 31.6 - 36.3 kg/m^2^) and morbidly obese patients’ BMI ranged 40.0-68.8 kg/m^2^ (IQR:41.9-49.7 kg/m^2^).

^a^Significantly difference between groups.

The primary composite endpoint occurred in 3.8% of patients taking apixaban and 1.7% of patients taking rivaroxaban (*P* = .28). TIA was the most frequently occurring event in both arms. No patient in the rivaroxaban arm had a stroke, MI, or atrial thrombus, while there was 1 of each event and more TIA occurrences in the apixaban arm. Bleeding events occurred in 2.8% of patients in the apixaban group compared with 1.7% in the rivaroxaban group (*P* = .5). Overall mortality was higher in those prescribed apixaban (3.8% vs 0%, *P* = .03). All endpoints are reported in Table 2.

**Table 2.** Primary Endpoint, Incidence of Bleeding Events, and All-Cause Mortality.

	Rivaroxaban (n = 121)	Apixaban (n = 212)	*P*-value
Primary composite (n, %)	2 (1.7%)	8 (3.8%)	.28
Atrial thrombosis	0 (0%)	1 (.5%)	—
MI	0 (0%)	1 (.5%)	—
Stroke	0 (0%)	1 (.5%)	—
TIA	2 (1.7%)	5 (2.4%)	—

Any bleeding event	2 (1.7%)	6 (2.8%)	.50
Clinically relevant, non-major bleeding (n, %)	2 (1.7%)	4, (1.9%)	—
Major bleeding (n, %)	0 (.0%)	2 (.9%)	—

Died (n, %)	0 (.0%)	8 (3.8%)	.03

Regression analysis results are shown in Table 3. No identified variables are significantly associated with the composite primary endpoint. Bleeding events were significantly associated with older age, male gender, CHA₂DS₂-VASc scores, and prior history of bleeds; when these variables were controlled for, the relationship between study arms and instances of bleeding remained non-significant. Mortality was significantly associated with clotting events.

**Table 3.** Regression Modeling of Parameters on Death, Bleeding, and Clotting Outcomes.

Outcome	Variable	Beta	*P*-value
Primary composite	Drinks alcohol	.20	.62
	HAS-BLED score	−.09	.69
	Renal disease	−.65	.61
	History of bleeding	−.32	.75
Any bleeding event	Drinks alcohol	−.23	.47
	HAS-BLED score	.20	.11
	Renal disease	.04	.64
	History of bleeding^a^	−2.39	<.01
	Age^a^	1.38	<.01
	Male^a^	−.53	.02
	CHA₂DS₂-VASc score^a^	.71	.03
Death	Drinks alcohol	.48	.36
	HAS-BLED score	.21	.58
	Renal disease	−1.12	.12
	History of bleeding	−.32	.47
	Any clotting event^a^	−2.79	.01

^a^Significantly associated with outcome.

## Discussion

Two phase III clinical trials examined the safety and efficacy of apixaban (ARISTOTLE) and rivaroxaban (ROCKET-AF) vs warfarin for the prevention of thromboembolic events in NVAF patients. Of the 2 trials, only ROCKET-AF included a prespecified subgroup analysis that included overweight and obese patients (BMIs ≥ 25 kg/m^2^ and weight ≥ 90 kg), for which there showed no significant interaction for BMI or weight.^1^ ARISTOTLE only provided a subgroup analysis on patients weighing ≥60 kg.^2^ A post-hoc analysis of the apixaban trial demonstrated that the efficacy of apixaban, compared to warfarin for the primary endpoint of stroke and systemic embolism, was not significantly impacted by the stratification of BMI.^15^ Despite these findings, no comparisons have been made between apixaban and rivaroxaban for obese patients with NVAF.

Real-world observational data for inter-class comparisons of DOACs for NVAF have been published in recent years, which include non-obese populations.^16–20^ Noseworthy and colleagues conducted a study using administrative claims data that included comparative cohorts of dabigatran, apixaban, and rivaroxaban-treated patients matched by propensity scores. In their comparison of apixaban and rivaroxaban, which included 13,130 patients, the rate of stroke or systemic embolism did not differ significantly (HR 1.05 [95% CI .64, 1.72]; *P* = .85); however, major bleeding was significantly less common with apixaban (HR .39 [95% CI .28, .54]; *P* < .001).^20^ No baseline characteristics regarding patient weight were provided. In a later study, Fralick and colleagues compared apixaban and rivaroxaban using propensity-matched cohorts, including 28.5% of overweight or obese patients (N = 78,702).^19^ Stroke and systemic embolism occurred at a rate of 6.6 vs 8.0 events per 1000 person-years for apixaban and rivaroxaban, respectively (HR .82 [95% CI .68, .98]). Bleeding was also less common with apixaban (HR .58 [95% CI .52, .66]).^19^ In addition to the real-world evidence above, 1 meta-analysis showed increased bleeding with rivaroxaban when examining complied outcomes from network meta-analyses.^16^ A sizable study including over 580,000 Medicare beneficiaries showed that the incidence of major ischemic or hemorrhagic events occurred less with apixaban than rivaroxaban (HR 1.18 [95% CI 1.12, 1.24]).^21^ These mentioned studies are compelling but are not specific to obese patient populations. Given that no differences in bleeding outcomes were seen between the 2 DOACs in our study, other factors may need to be considered. A numerically higher rate of bleeding with apixaban was seen in our patient sample, which is surprising considering altered PK parameters of apixaban that should theoretically reduce bleeding events. One explanation for a higher bleeding event rate in the apixaban arm could be baseline characteristics imbalances in prior bleeding events and alcohol use, which both impacted HAS-BLED scores.

Observational studies providing an intercomparison of DOACs specifically in obese NVAF patients are limited. Kushnir and colleagues evaluated 429 morbidly obese patients (BMI ≥ 40 kg/m^2^) prescribed anticoagulation for NVAF at a single-center and found no significant differences in stroke rate with a three-way comparison between apixaban, rivaroxaban, and warfarin (*P* = .71).^6^ Another study comparing DOACs collectively (apixaban, rivaroxaban, and dabigatran) to warfarin in morbidly obese NVAF patients observed similar rates of ischemic stroke and TIA 1.75%/year vs 2.07%/year (RR .84 [95% CI .23, 3.14]; *P* = .8).^5^ Although no direct comparisons between rivaroxaban and apixaban were made, 1.07%/year experienced stroke or TIA with rivaroxaban and no events occurred for patients on apixaban. In a more recent study by Briasoulis and colleagues, obese and morbidly obese patients (N = 28,011) receiving apixaban, rivaroxaban, dabigatran, and warfarin in the Veterans Health Administration system were examined.^22^ Apixaban was found to have significantly higher ischemic stroke risk and all-cause mortality when compared with rivaroxaban (*P* < .001 for both); however, apixaban was associated with significantly lower major bleeding risk compared to rivaroxaban (*P* < .001).^22^ While our study did not show any difference between bleeding and thrombosis events, similar to the Kushnir and Kido studies, we did see a difference in overall mortality rate in favor of rivaroxaban.

Current recommendations to use any DOAC in class I and II obesity are based on indirect comparisons between phase III sub-group analyses and post-hoc studies.^23^ The American Heart Association/American College of Cardiology/Heart Rhythm Society guidelines recognize that DOAC serum levels are available and may be used to evaluate drug absorption in severely obese patients defined as BMI > 35 or weight > 120 kg; however, there are limited data supporting the correlations between these levels and clinical outcomes.^3^ Based on our study and previous studies, there is a possibility that obese patients have worse outcomes with conventional apixaban dosing. Until more robust safety and efficacy data become available, a patient-centered approach considering bleeding and thrombotic risks, with potential monitoring, and evaluation of medication access concerns, should be utilized in the obese patient population.

Several limitations of this study should be noted. First, the sample population includes patients treated at a single cardiovascular clinic in East Texas, which is >90% Caucasian. Due to the study's retrospective nature, the investigators relied on outcome data being present in the shared electronic health record of the clinic’s associated health-system. Prescription claims data was not assessed, and therefore compliance information for the prescribed NOAC is not presented. Finally, the observed event rate was small, and despite including all eligible patients, adequate power may not be met. Power was not calculated due to all eligible patients being included, but based on the anticipated frequency of events, it is likely that the study is underpowered.

Strengths of the study should also be mentioned. Our study had a high proportion of identified patients that met inclusion and having records from an outpatient clinic and hospitalizations allowed for comprehensive oversight of the included patients; however, the sample size is relatively small. Additionally, all authors were involved in data validation for all primary and secondary endpoints, minimizing erroneous outcomes. As with any retrospective observational study, only associations can be identified and results do not imply causality.

## Conclusions

Among obese patients treated with apixaban or rivaroxaban at a single outpatient clinic, the rates of stroke, TIA, MI, and atrial thrombosis did not differ. Despite prior pharmacokinetic data, the apixaban arm also experienced more bleeding episodes, but the difference was not significant, and differences in baseline bleed risk need to be accounted for. Until more evidence becomes available, a patient-centered approach considering all risks and benefits should be employed when determining the best anticoagulant to use. Future studies should continue to conduct intercomparison between these DOACs in obese patients or include a predefined subgroup analysis.
